# The influence of anatase-rutile mixed phase and ZnO blocking layer on dye-sensitized solar cells based on TiO_2_nanofiberphotoanodes

**DOI:** 10.1186/1556-276X-8-9

**Published:** 2013-01-03

**Authors:** Jianning Ding, Yan Li, Hongwei Hu, Li Bai, Shuai Zhang, Ningyi Yuan

**Affiliations:** 1 Center for Low-dimensional Materials, Micro-nano Devices and System, Changzhou University, Changzhou 213164, China; 2 Jiangsu Key Laboratory for Solar Cell Materials and Technology, Changzhou 213164, China

**Keywords:** Dye-sensitized solar cell, Titanium dioxide nanofiber photoanode, Anatase-rutile mixed phase, Zinc oxide blocking layer, Atomic layer deposition method

## Abstract

High performance is expected in dye-sensitized solar cells (DSSCs) that utilize one-dimensional (1-D) TiO_2_ nanostructures owing to the effective electron transport. However, due to the low dye adsorption, mainly because of their smooth surfaces, 1-D TiO_2_ DSSCs show relatively lower efficiencies than nanoparticle-based ones. Herein, we demonstrate a very simple approach using thick TiO_2_ electrospun nanofiber films as photoanodes to obtain high conversion efficiency. To improve the performance of the DSCCs, anatase-rutile mixed-phase TiO_2_ nanofibers are achieved by increasing sintering temperature above 500°C, and very thin ZnO films are deposited by atomic layer deposition (ALD) method as blocking layers. With approximately 40-μm-thick mixed-phase (approximately 15.6 wt.% rutile) TiO_2_ nanofiber as photoanode and 15-nm-thick compact ZnO film as a blocking layer in DSSC, the photoelectric conversion efficiency and short-circuit current are measured as 8.01% and 17.3 mA cm^−2^, respectively. Intensity-modulated photocurrent spectroscopy and intensity-modulated photovoltage spectroscopy measurements reveal that extremely large electron diffusion length is the key point to support the usage of thick TiO_2_ nanofibers as photoanodes with very thin ZnO blocking layers to obtain high photocurrents and high conversion efficiencies.

## Background

Due to their cost-effectiveness, ease of manufacturing, and suitability for large-area production, dye-sensitized solar cells (DSSCs) have attracted much attention. Typically, the photoanode of a DSSC is made of a TiO_2_ nanoparticle film (10-μm thickness) adsorbed with a monolayer Ru-based complex dye. Although the certified energy conversion efficiency of DSSCs has exceeded 12% [1], electrons generated from photoexcited dyes injected into the conduction band of TiO_2_ will pass through the grain boundaries and interparticle connections, which are strongly influenced by the surface trapping/detrapping effect, leading to slow electron transport [2]. One-dimensional (1-D) nanostructures have superior electron transport characteristics compared to nanoparticle-based systems [3,4]. Several methods have been established for the preparation of 1-D structured TiO_2_, including nanowires [5,6], nanotubes [7-10] and nanofibers. Among the methods for preparing 1-D TiO_2_ nanostructures, electrospinning provides a versatile, simple, and continuous process [11-13]. However, even though extremely fast electron transport is available in the 1-D nanostructures, these 1-D TiO_2_-based DSSCs usually show relatively lower efficiencies than nanoparticle-based ones, mainly because of low dye adsorption. To solve this problem of TiO_2_ electrospun nanofiber DSSCs, some attempts have been done, such as applying mechanical pressure to break the outer sheaths of nanofibers to increase surface area [14,15], calcination of nanofibers with a hot pressing pre-treatment to obtain multi-core cable-like nanofibers [16]. However, these methods destroy continuous 1-D nanostructures. In view of the excellent electron transport characteristic, which will result in a large diffusion length, it is feasible to increase the thickness of 1-D nanostructure photoanodes to improve dye adsorption and, consequently, to enhance the conversion efficiency of cells. Unfortunately, the lengths of TiO_2_ nanowires or nanorods are usually several micrometers [5,6], and it is a very difficult or time-consuming mission to enlarge their length, so the conversion efficiency is limited. Long TiO_2_ nanotube can be formed by anodization of titanium foils [17]. However, backside-illumination mode of anodized TiO_2_ nanotube-based solar cells is an obstacle for realizing a high efficiency since the redox electrolyte containing the iodine species has an absorption in near UV spectrum and platinum-coated fluorine-doped SnO_2_ (FTO) partially and inevitably reflects light [17,18]. On the contrary, it is very easy within a short period of process to enlarge the thickness of TiO_2_ electrospun nanofiber photoanode on FTO substrates for front illumination.

On the other hand, superior performance of anatase-rutile mixed-phase TiO_2_ nanoparticle DSSCs with a small amount of rutile to pure phase ones was claimed [19,20]. Different from nanoparticles, it is relatively difficult for nanowires or nanotubes to control their crystalline phase, so there are little researches on anatase-rutile mixed-phase 1-D TiO_2_ DSSCs. Besides, it has been proven effective to block electron recombination by introduction of a compact layer, such as TiO_2_[21-25], Nb_2_O_5_[26], and ZnO [27,28] between the FTO and porous TiO_2_. Nb_2_O_5_ is an expensive material for compact film. For ZnO, not only electron transmission is faster than that in TiO_2_ but also its conduction band edge is a little more negative than that of TiO_2_, which will introduce an energy barrier at the interface of FTO/TiO_2_. The energy barrier will be favorable to suppress the back electron transfer from FTO to electrolytes. However, the thickness of the reported ZnO blocking layers deposited by sputtering methods [27,28] was around 150 nm to get the highest conversion efficiency. Thick blocking layers will reduce transmittance of FTO substrates and consequently decrease the absorption of visible light. Meanwhile, it probably retards the transport of injected electrons from TiO_2_ conduction band to FTO, resulting in a low photocurrent [28]. Atomic layer deposition (ALD) technique can produce continuous, angstrom-level-controlled, and defect-free films, which is very suitable to deposit ultrathin compact film.

In this paper, to make the best of excellent electron transport characteristic of 1-D nanostructures, thick TiO_2_ nanofiber films were used as photoanodes to fabricate DSSCs. Meanwhile, anatase-rutile mixed-phase TiO_2_ nanofibers obtained by increasing sintering temperature and very thin ZnO compact layers deposited by ALD method were first adopted in the TiO_2_ nanofiber DSSC fabrication to further improve photocurrent and conversion efficiency. Combining the above two steps, a short-circuit current density of 17.3 mAcm^−2^ and a conversion efficiency of 8.01% were achieved for the DSSC using approximately 40-μm-thick TiO_2_ nanofiber film as photoanode. Intensity-modulated photocurrent spectroscopy (IMPS) and intensity-modulated photovoltage spectroscopy (IMVS) were used to investigate the dynamic response of charge transfer and recombination in TiO_2_ nanofiber DSSCs.

## Methods

### TiO_2_ nanofiber synthesis

The polyvinylpyrrolidone (PVP)-TiO_2_ nanofibers were fabricated using electrospinning technique. Typically, the precursor solution for electrospinning was made from 0.45 g of PVP (with a molecular weight of 1,300,000; Sigma-Aldrich Corporation, St. Louis, MO, USA), 7 ml of ethanol, 2 ml of acetic acid, and 1 g of titanium (IV) isopropoxide (Sigma-Aldrich). In a typical electrospinning procedure, the precursor solution was loaded into a syringe equipped with a 24 gauge silver-coated needle. The needle was connected to a high-voltage power supply. The electric voltage of 16 kV was applied between the metal orifice and the Al collector at a distance of 10 cm. The spinning rate was controlled by the syringe pump at 60 μl min^−1^. After the electrospinning procedure, the PVP-TiO_2_ fiber composite films were then heated at a rate of 4°C min^−1^ up to 500°C, 550°C, 600°C, and 700°C, respectively, and then sintered at this temperature for 2 h to obtain pure TiO_2_-based nanofibers.

### Preparation of ultrathin ZnO blocking layers by ALD method

ZnO layers were deposited on FTO-coated glass substrates (25 Ω/sq) by ALD method. FTO glass plates were first cleaned in a detergent solution using an ultrasonic bath for 15 min and were then rinsed with water and ethanol. Diethylzinc (DEZ; Zn(C_2_H_5_)_2_) and deionized water were used as precursors for ZnO deposition on the cleaned FTO plates. Pure N_2_ gas (99.999%) was used to carry and purge gas. The reaction was carried out as follows:

(1) $$\mathrm{Zn}{\left(C_{2}H_{5}\right)}_{2}+H_{2}O\to {\mathrm{Zn}\;O+2C}_{2}H_{6}$$

Before deposition, the reaction chamber was pumped down from 1 to 2 Torr. The operating environment of ZnO deposition was maintained at 3 Torr and 200°C. Each deposition cycle consisted of four steps, which included DEZ reactant, N_2_ purge, H_2_O reactant, and N_2_ purge. The typical pulse time for introducing DEZ and H_2_O precursors was 0.5 s, and the purge time of N_2_ was 10 s. The deposition rate of ZnO film at the above conditions approached 0.182 nm/cycle. Thus, the deposition cycles of 22, 55, 83, and 110 were chosen to produce ZnO layers with thicknesses of 4, 10, 15, and 20 nm.

### Solar cell fabrication and characterization

First, the substrates were coated with TiO_2_ nanofiber films using a mixed solution (20 ml ethanol and 2 ml titanium (IV) isopropoxide) for enhancing the adhesion between TiO_2_ films and substrates, and were subsequently sintered at 500°C for 30 min to ensure good electrical contact between the TiO_2_ nanofiber films and the FTO substrates. Then, the TiO_2_ electrodes were immersed into the N-719 dye solution (0.5 mM in ethanol) and were held at room temperature for 24 h. The dye-treated TiO_2_ electrodes were rinsed with ethanol and dried under nitrogen flow. For the counter electrodes, the FTO plates were drilled and coated with a drop of 10 mM H_2_PtC_l6_ (99.99%, Sigma-Aldrich) solution and were then heated at 400°C for 20 min. The liquid electrolyte was prepared by dissolving 0.6 M of 1-butyl-3-methylimidazolium iodide, 0.03 M of iodine, 0.1 M of guanidinium thiocyanate, and 0.5 M of 4-*tert*-butylpyridine in acetonitrile/valeronitrile (85:15 *v*/*v*). Finally, dye-coated TiO_2_ films and Pt counter electrodes were assembled into sealed sandwich-type cells by heating with hot-melt films used as spacers. The typical active area of the cell was 0.25 cm^2^.

The crystallographic structure of the nanofiber was analyzed by X-ray diffraction (XRD) (D/MAX Ultima III, Rigaku Corporation, Tokyo, Japan) using Cu Kα radiation. The morphology was determined by scanning electron microscopy (SEM). Specific surface areas of the nanofibers in powder form were measured with a Quantachrome Autosorb-3b static volumetric instrument (Quantachrome Instruments, Boynton Beach, FL, USA). UV-visible (UV–vis) spectra were carried out on a Hitachi U-3010 spectrophotometer (Hitachi, Ltd., Chiyoda, Tokyo, Japan). The thicknesses of the films were obtained using an α-Step 500 surface-profile measurement system (KLA-Tencor Corporation, Milpitas, CA, USA). Photovoltaic characteristics were measured using a Keithley 2400 source meter (Keithley Instruments Inc., Cleveland, OH, USA). A solar simulator (500-W Xe lamp) was employed as the light source, and the light intensity was adjusted with a Si reference solar cell for approximating AM 1.5 global radiation. IMPS and IMVS spectra were measured on a controlled intensity-modulated photospectroscopy (Zahner Co., Kansas City, MO, USA) in ambient conditions under illumination through the FTO glass side, using a blue light-emitting diode as the light source (BLL01, *λ*_max_ = 470 nm, spectral half-width = 25 nm; Zahner Co.) driven by a frequency response analyzer, and the light intensity (incident photon flux) of the DC component was controlled at 2.5 × 10^16^ cm^−2^ s^−1^. During the IMVS and IMPS measurements, the cell was illuminated with sinusoidally modulated light having a small AC component (10% or less of the DC component).

## Results and discussion

### Characterization of TiO_2_ nanofibers

**Figure 1:**
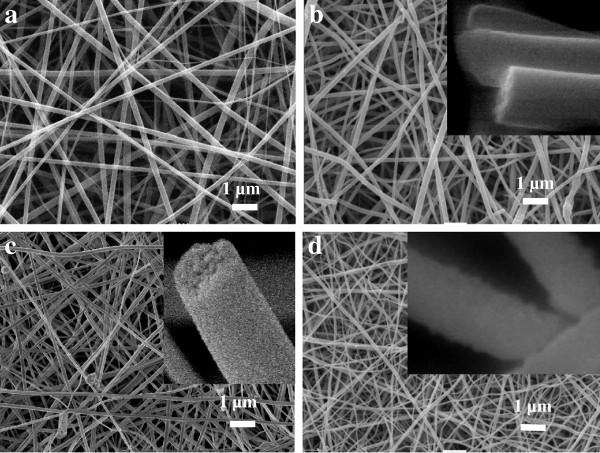
**SEM images of electrospun nanofibers.** As-spun TiO_2_-PVP nanofibers (**a**), TiO_2_ nanofibers after calcination at 500°C (**b**), 550°C (**c**), and 600°C (**d**). The insets in b, c, and d are high-magnification photos of single nanofibers.

**Figure 2:**
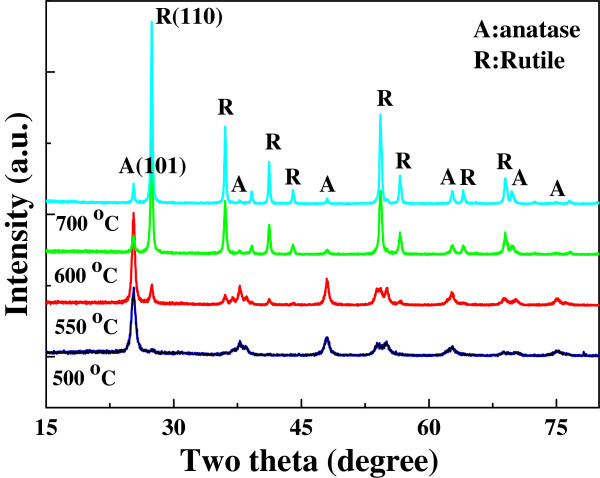
**XRD patterns of TiO**_**2**_**nanofibers sintered at 500°C, 550°C, 600°C, and 700°C.** The diffractions of anatase and rutile phase are labeled in the figure as ‘A’ and ‘R’, respectively.

The surface morphologies of as-spun TiO_2_-PVP composite and sintered TiO_2_ nanofibers were characterized by SEM as shown in Figure 1. It is found that the network structure of the former is maintained after calcinations in air to remove PVP, forming a porous TiO_2_ membrane. The higher sintering temperature, the thinner is TiO_2_ fiber. The average fiber diameter of the composite nanofibers is 290 ± 90 nm which decreases to 210 ± 60 nm, 180 ± 70 nm, and 140 ± 80 nm after sintering at 500°C, 550°C, and 600°C, respectively. It is known that crystalline grains of anatase TiO_2_ are spherical, while rutile ones are of rod structure. With the increase of the sintering temperature, some anatase TiO_2_ grains will transform to rutile ones, which may result in the thinning of the fibers. Moreover, transformation of anatase TiO_2_ grains to rutile ones will introduce stress in the fibers, which will cause the fibers to become brittle and even fracture. The insets in Figure 1b, c, d are high-magnification photos of nanofibers, which indicate that the surfaces of TiO_2_ nanofibers sintered at 500°C and 550°C are rather smooth, while become a little rough when sintering temperature increases to 600°C. Figure 2 shows the XRD patterns of TiO_2_ nanofibers. All the peaks of the TiO_2_ nanofibers sintered at 500°C are indexed for anatase TiO_2_ with dominant (101) peaks. The mean grain size determined from the XRD pattern using the Scherrer formula is around 16 nm. The nanofibers sintered at 550°C, 600°C, and 700°C are observed to contain both anatase and rutile phases. The phase composition can be determined from XRD results according to the following equation [29]:

(2) $$W_{R}=A_{R}/\left(0.884\times A_{A}+A_{R}\right)$$

where *W*_R_, *A*_A_, and *A*_R_ represent rutile weight percentage, integrated intensity of anatase (101) peak, and rutile (110) peak, respectively [29]. The calculated rutile contents in the above three mixed-phase nanofiber samples are approximately 15.6, 87.8, and 90.5 wt.%, and the mean grain sizes are 22, 30, and 42 nm, respectively. The XRD results indicate that with the increase of sintering temperature, the grain size is gradually increased; however, rutile content is sharply increased in the temperature range of 550°C to 600°C.

### Characterization of ultrathin ZnO layers deposited by ALD method

**Figure 3:**
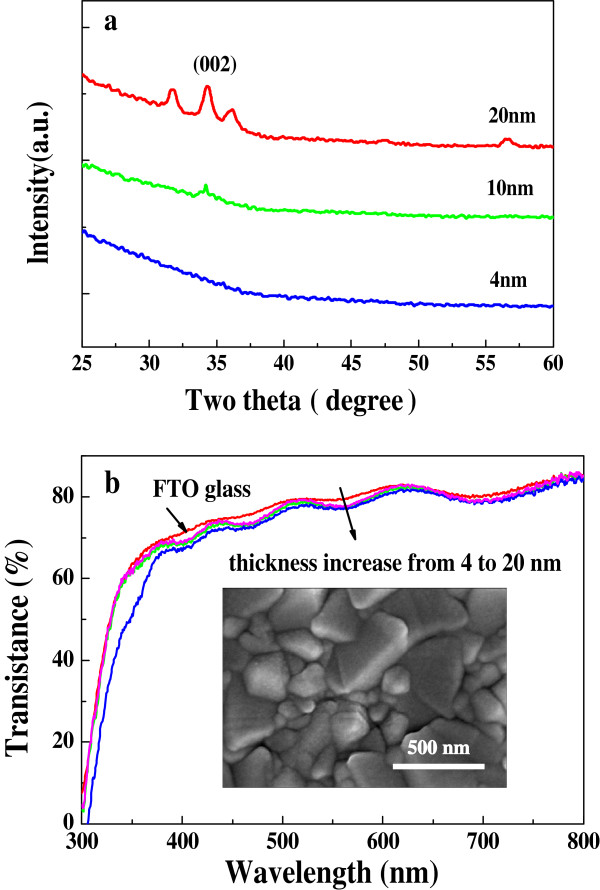
**XRD patterns.** ZnO layers deposited on glass substrate (**a**) and UV–vis transmission spectra for the FTO substrate without and with ZnO layers (**b**). The inset in b is the SEM photo of FTO substrate covered with 15-nm-thick ZnO film.

To detect the crystallographic structure and thickness of ZnO layers, except FTO substrates, glass substrates were also used to deposit ZnO layers. XRD patterns for ZnO layers deposited on glass substrate are shown in Figure 3a. A 4-nm-thick ZnO layer does not show any diffraction peak, whereas peaks corresponding to hexagonal phase ZnO are observed for the thickness of 10 or 20 nm, which indicates that the deposited ZnO layers by ALD method are polycrystalline. Figure 3b shows the UV–vis transmission spectra for the FTO substrates without ZnO layers and with ZnO layers of different thicknesses. The absorbance edges for the bare FTO and ZnO are around 315 and 380 nm, respectively, from which the bandgap of ZnO is calculated to be 3.2 to 3.3 eV. In the visible range, the transmittance of the FTO covered with ZnO decreases slightly with the increase of ZnO film thickness. For instance, it decreases to approximately 95% of the transmittance of the bare FTO for 20-nm-thick ZnO. Therefore, the presence of ZnO layer with the thickness less than 20 nm will not obviously influence the harvest of light. The inset in Figure 3b is the SEM photo of FTO substrate covered with 15-nm-thick ZnO film, which shows that the ZnO film deposited by ALD method keeps the surface morphology of FTO substrate very well.

### Performance of DSSCs

#### The influence of sintering temperature of TiO_2_ nanofiber photoanodes on the performance of TiO_2_ nanofiber cells

**Figure 4:**
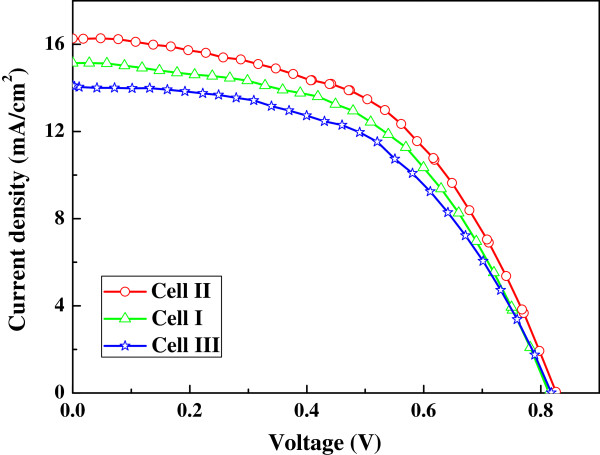
**Photocurrent-voltage characteristics of cells I to III under AM 1.5 irradiation of 100 mW cm**^−**2**^**.** Based on TiO_2_ nanofibers sintered at 500°C, 550°C, and 600°C.

Cells I to III are TiO_2_ nanofiber cells (sintered at 500°C, 550°C, and 600°C) on the bare FTO substrates. Based on the above photocurrent-voltage(*JV*) measurement results, a thickness of approximately 40 μm was set to fabricate cells I to III. Figure 4 illustrates the *JV* characteristics of TiO_2_ nanofiber cells under AM 1.5 irradiation of 100 mW cm^−2^. The photovoltaic properties such as short-circuit current density (*J*_sc_), open-circuit voltage (*V*_oc_), fill factor (FF), and photoelectric conversion efficiency (PCE) of the cells are listed in Table 1. Cell I has a *J*_sc_ of 15.1 mA cm^−2^, PCE of 6.39%, *V*_oc_ of 0.814 V, and fill factor (FF) of 0.52. When sintering temperature increased from 500°C to 550°C, cell II gave an improvement of *J*_sc_ and *V*_oc_ about 1.2 mA cm^−2^ and 11 mV, respectively, resulting in an efficiency of 7.12%. However, the further increase of sintering temperature decreased *J*_sc_, *V*_oc_, and PCE of cell III to 14.1 mA cm^−2^, 0.818 V, and 6.11%, respectively. According to the XRD data, rutile contents of TiO_2_ nanofibers are approximately 0, 15.6, and 87.8 wt.% in cells I, II, and III, respectively. The *JV* measurement results demonstrate that the anatase-rutile mixed-phase TiO_2_ nanofiber with a low rutile content is good for enhancing efficiencies of the DSSCs, whereas a high rutile content is detrimental to the efficiencies, which is similar to the reported DSSCs based on mixed-phase TiO_2_ nanoparticles [19,20].

**Table 1:** **Photocurrent density-voltage characteristics of TiO**_**2**_**nanofiber cells sintered at 500°C, 550°C, and 600°C**

**Cell**	**Temperature (°C)**	***J***_**sc**_**(mA/cm**^**2**^**)**	***V***_**oc**_**(V)**	**FF**	***η *(%)**	***τ***_**d**_**(ms)**	***τ***_**n**_**(ms)**	***L***_**n**_**(μm)**
I	500	15.1	0.814	0.52	6.39	3.36	55.3	74.2
II	550	16.3	0.825	0.53	7.12	1.88	107.7	138.3
III	600	14.1	0.818	0.53	6.11	6.47	86.9	67.1

TiO_2_ nanofiber cells on the bare FTO substrates, the transit time (*τ*_d_) and electron lifetime (*τ*_n_), and diffusion length (*L*_n_).

**Figure 5:**
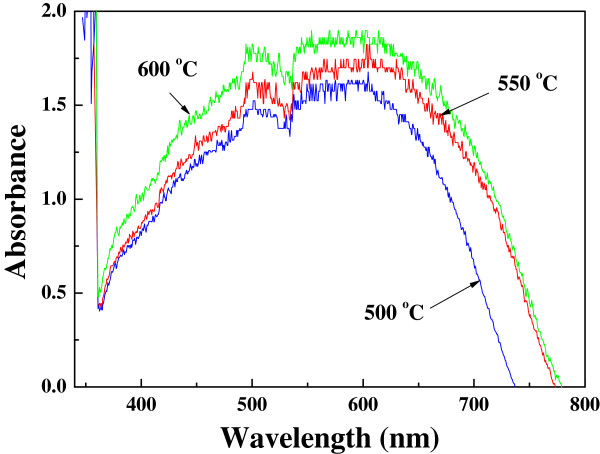
**UV–vis absorption spectra.** Sensitized TiO_2_ nanofiber films (approximately 60-μm thick) sintered at 500°C, 550°C, and 600°C.

In this study, specific surface areas were measured to be 28.5, 31.7, and 34.2 m^2^ g^−1^ for TiO_2_ nanofibers sintered at 500°C, 550°C, and 600°C, respectively, which indicate that thinner rough nanofibers sintered at a higher temperature is favorable to increase the specific surface areas. UV–vis absorption spectra (Figure 5) of the sensitized TiO_2_ nanofiber film show that the absorption edges are successfully extended to the visible region for all the three samples. In contrast with pure anatase phase (sintered at 500°C), mixed-phase TiO_2_ nanofibers (sintered at 550°C and 600°C) after N719 sensitization absorb a greater portion of the visible light, which should be the result of joint contribution of large specific surface area and mixed phase. Because anatase phase TiO_2_ has the greatest dye absorption ability, while rutile phase TiO_2_ possesses excellent light scattering characteristics due to its high refractive index (*n* = 2.7) [25,26], dye-sensitized anatase-rutile mixed-phase TiO_2_ with a proper proportion will have an enhanced light absorption.

**Figure 6:**
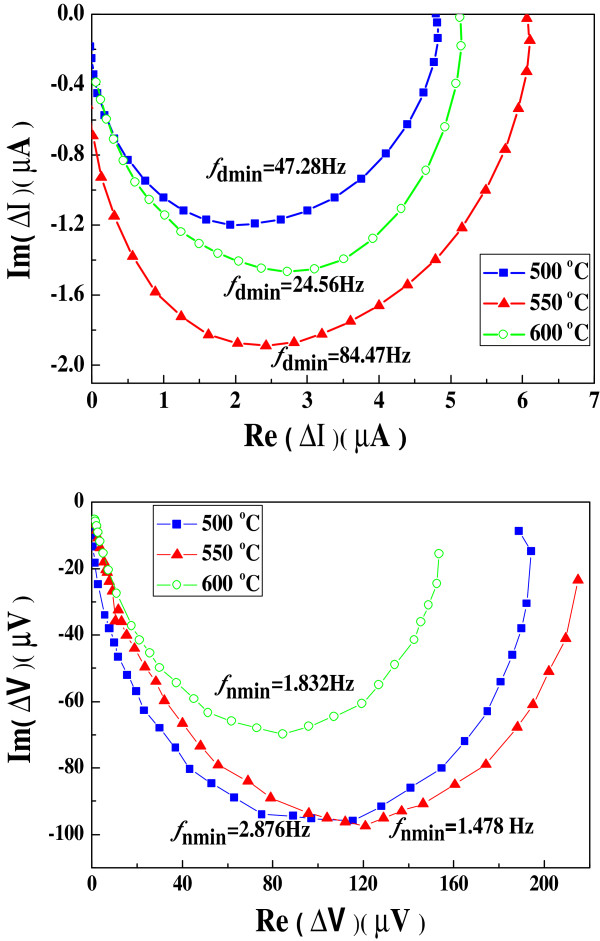
**IMPS (a) and IMVS (b) plots of cells I to III.** Based on TiO_2_ nanofibers sintered at 500°C, 550°C, and 600°C.

The IMPS and IMVS plots of cells I to III display semicircles in the complex plane as shown in Figure 6. The transit time (*τ*_d_) and electron lifetime (*τ*_n_) can be calculated using the equations *τ*_d_ = 1/(2π*f*_IMPS__min_) and *τ*_n_ = 1/(2π*f*_IMVS,min_), respectively, where *f*_IMPS,min_ and *f*_IMVS,min_ are the frequencies at the minimum imaginary component in the IMPS and IMVS plots [30]. The estimated electron lifetimes of the three cells follow the trend *τ*_n II_ >*τ*_n III_ >*τ*_n I_, suggesting a reduction in recombination of electrons at the interface between TiO_2_ and electrolyte in the presence of rutile phase, while transit times vary in the order *τ*_d II_ >*τ*_d I_ >*τ*_d III_, indicating that the variation in electron transport rate is dependent on the amount of rutile phase. The competition between collection and recombination of electrons can be expressed in terms of the electron diffusion length. The electron collection efficiency is determined by the effective electron diffusion length, *L*_n_, [31]:

(3) $$L_{n}=d{\left(\tau_{n}/2.35\tau_{d}\right)}^{1/2}$$

where *d* is the thickness of the photoanode. The calculated *L*_n_/*d* (as shown in Table 1) of TiO_2_ nanofiber cell is large and follows the sequence *L*_n II_/*d*_II_ >*L*_n I_/*d*_I_ >*L*_n III_/*d*_III_. A remarkable large value of 4.9 is found for cell II. A large electron diffusion length is the key point to support the usage of thick TiO_2_nanofibers as photoanodes to obtain high photocurrents and high conversion efficiencies. The largest *L*_n_/*d*_II_ of cell II with 15.6% rutile content is speculated to be caused by the interaction of rutile and anatase phases which prompts electron transfer from anatase to rutile due to their different conduction band edge and decreased charge recombination rate. However, if the amount of rutile phase is too high in TiO_2_ nanofibers, such as 87.8% in cell III, the property of rutile phase will play a leading role in the cell. A large transit time shows a slow electron transport in cell III, which leads to a decrease in electron diffusion length for cell III. From the above analysis, it is concluded that the superior *J*_sc_ of cell II is a consequence of more efficient electron collection and light harvesting. As far as *V*_oc_ is concerned, it is known that *V*_oc_ corresponds to the energy difference between the quasi-Fermi level of the electrons in the TiO_2_ under illumination and the redox potential. If the electron recombination is retarded, the electron density in the conduction band of TiO_2_ will be increased, which will result in a negative shift in quasi-Fermi level, thereby *V*_oc_ will be increased [32]. Thus, the higher *V*_oc_ of cell II is ascribed to the reduced electron recombination rate. For cell III, in spite of the largest absorbance of visible light, a relatively low *J*_sc_ is produced because of an inefficient electron collection. The comparison of cells I to III highlights the existence of a synergistic effect between the anatase and rutile phases in TiO_2_ nanofiber DSSCs, as well as suggests a sintering temperature of approximately 550°C which is optimal for enhancing the performance of nanofiber DSSCs.

#### The influence of ZnO blocking layer on the performance of TiO_2_ nanofiber cells

**Figure 7:**
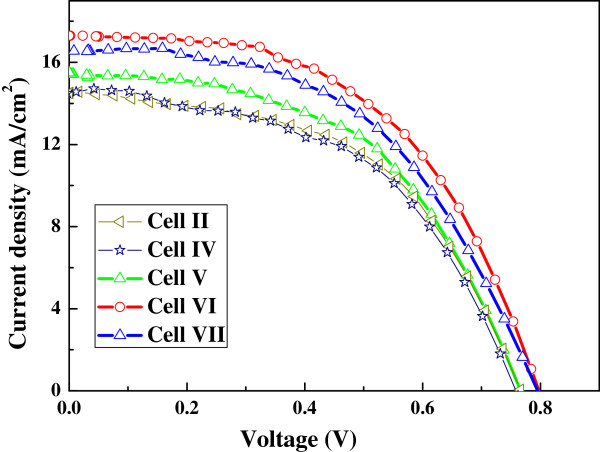
**Photocurrent-voltage curves of TiO**_**2**_**nanofiber cells (sintered at 550°C and approximately 60-μm thick).** With ZnO layer of different thicknesses (4 to 20 nm).

Based on the above results, cell II was chosen as the reference cell to study the influence of ZnO blocking layer on the performance of TiO_2_ nanofiber cells. ZnO layers with thicknesses of 4, 10, 15, and 20 nm were deposited by ALD method on FTO substrates to fabricate cells IV, V, VI, and VII, respectively. *JV* curves of cells II and IV to VII are shown in Figure 7, and the photovoltaic characteristics of these cells are summarized in Table 2. Compared with cell II, the performances of the cells with the ZnO layer are significantly improved. With the ZnO layer thickness increased from 0 to 15 nm, *J*_sc_ of the cells is monotonously boosted, but when decreased obviously at 20 nm, it is still larger than that without the ZnO layer. It is noticed that enhancement in *V*_oc_ and FF is very small. The largest *J*_sc_ of 17.3 mA cm^−2^ is obtained from cell VI with 15-nm-thick ZnO layer, resulting in the highest PCE of 8.01%, in contrast with 16.3 mA cm^−2^ and 7.12% of reference cell II. This phenomenon indicates that the charge collection of the cells is improved by the blocking function of ZnO layer on interfacial recombination, which is very different from the reported decrease of *J*_sc_ caused by thick ZnO blocking layers [30].

**Table 2:** **Photocurrent density-voltage characteristics of TiO**_**2**_**nanofiber cells**

**Cell**	**ZnO thickness (nm)**	***J***_**sc**_**(mA/cm**^**2**^**)**	***V***_**oc**_**(V)**	**FF**	***η *(%)**	***τ***_**d**_**(ms)**	***τ***_**n**_**(ms)**	***L***_**n**_**(μm)**
II	0	14.5	0.825	0.53	6.34	1.88	107.7	138.3
IV	4	15.0	0.828	0.54	6.71	1.43	119.5	166.9
V	10	16.5	0.833	0.54	7.42	1.21	154.3	206.4
VI	15	17.3	0.842	0.55	8.01	1.08	179.7	235.7
VII	20	14.8	0.825	0.53	6.47	4.62	354.5	159.9

TiO_2_ nanofiber cells with ZnO layer of different thicknesses, the transit time (*τ*_d_) and electron lifetime (*τ*_n_), and diffusion length (*L*_n_).

**Figure 8:**
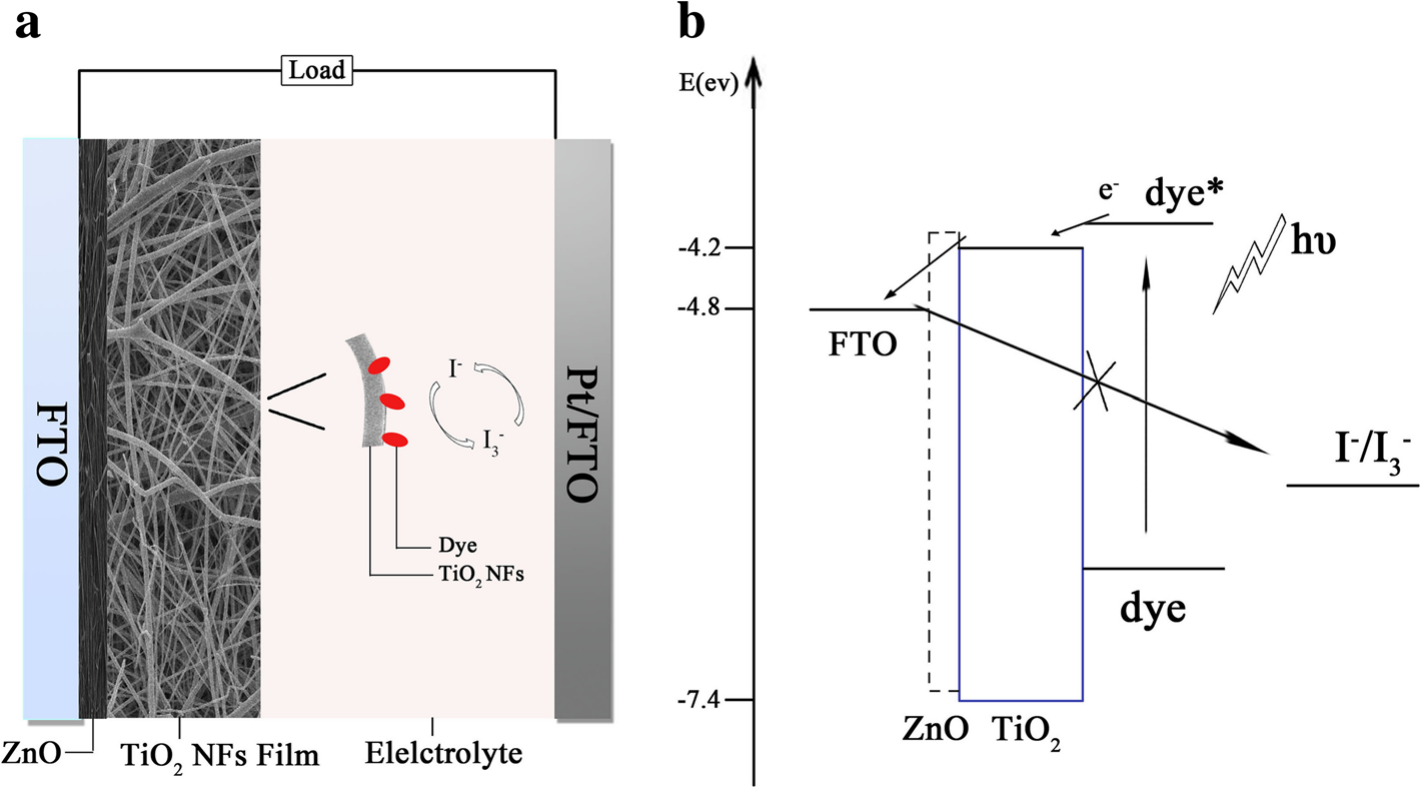
**Schematic view of electron transfer with ZnO layer.** TiO_2_ nanofiber DSSC with an ultrathin ZnO layer (**a**). Illustration of the interfacial charge-transfer processes occurring in the DSSC (**b**). Also shown is the blocking function of ZnO blocking layer on interfacial recombination as described in this paper.

The schematic view of electron transfer with ZnO layer is shown in Figure 8. The interfacial processes involved in charge transportation in the cell are depicted in Figure 8b. As exciton dissociation occurs, electrons injected into the TiO_2_ conduction band will transport to the FTO by diffusion [33]. Because the conduction band edge of ZnO is a little more negative than that of TiO_2_, an energy barrier is introduced at the interface of FTO/TiO_2_, in which ultrathin ZnO layer can effectively suppress the back electron transfer from FTO to electrolytes or may block injected electron transfer from TiO_2_ to FTO. The back reaction was studied using IMVS measurements. The electron lifetime *τ*_n_ obtained from IMVS (as shown in Table 2) is 107.7 ms for the cell without ZnO layer but is significantly increased from 119.5 to 354.5 ms with ZnO layer thickness increasing from 4 to 20 nm. The striking increase in the lifetime shows direct evidence that ultrathin ZnO layers prepared by ALD method successfully suppress the charge recombination between electrons emanating from the FTO substrate and I_3_^−^ ions in the electrolyte. The transit times of electrons calculated from IMPS measurements reflect charge transport and back reaction. Although an energy barrier is induced by introduction of ZnO layer between the TiO_2_ and FTO, the electron transit time estimated from IMPS measurement is decreased from 1.88 to 1.08 ms for cells with ZnO layer thickness increasing from 0 to 15 nm. However, when the thickness of ZnO layer further increases, the change trend is reverse, and electron transit time for the cell with 20-nm-thick ZnO layer is markedly increased to 4.62 ms. It is put forward that relative to the cell without ZnO blocking layer, the electron transport in the cells with ZnO layers is determined by the two competition roles of the suppression effect of recombination with I_3_^−^ and potential barrier blocking effect. The increased electron lifetime has verified that ultrathin ZnO layer effectively slows the back recombination of electrons at the interface of FTO/electrolyte, so the decreased electron transit time reveals that the suppression effect is stronger than the potential barrier effect when the ZnO layer thickness is smaller than 20 nm. The obtained values of *L*_n_/*d* of cells IV to VII are shown in Table 2, which are all larger than that of the reference cell without ZnO layer, with the largest value of 8.4 for cell VI with a 15-nm-thick ZnO blocking layer. As a consequence, *J*_sc_'s of the four cells are significantly improved and reaches the largest value of 17.3 mA cm^−2^ for cell VI. No matter significant improvement of *J*_sc_'s for the four cells, little variation in *V*_oc_ is found for cells with and without ZnO layers, manifesting no electrons accumulate at the interface between ZnO and TiO_2_, which is in good agreement with the rapid transport of injected electrons in TiO_2_ conduction band to FTO substrates through ZnO layers.

## Conclusions

In summary, thick electrospun TiO_2_ nanofibers sintered at 500°C to 600°C were used as photoanodes to fabricate DSSCs. The remarkable electron diffusion length in TiO_2_ nanofiber cells is the key point that makes it feasible to use thick photoanode to obtain high photocurrent and high conversion efficiency. Besides, at sintering temperature of 550°C, a small rutile content in the nanofiber (approximately 15.6%) improved conversion efficiency, short-circuit current, and open-circuit voltage of the cell by 10.9%, 7.4%, and 1.35%, respectively. Moreover, it is demonstrated that ultrathin ZnO layer prepared by ALD method could effectively suppress the electron transfer from FTO to electrolytes by IMVS measurements, and its suppression effect of back reaction was stronger than the potential barrier effect of electron transfer from TiO_2_ to FTO by IMPS measurements. A large ratio of electron diffusion length to photoanode thickness (*L*_n_/*d*) was obtained in the approximately 40-μm-thick TiO_2_ nanofiber DSSC with a 15-nm-thick ZnO blocking layer, which is responsible for short-circuit current density of 17.3 mA cm^−2^ and conversion efficiency of 8.01%. The research provides a potential approach to fabricate high-efficient DSSCs.

## Abbreviations

ALD: atomic layer deposition; DSSCs: dye-sensitized solar cells; FF: fill factor; FTO: fluorine-doped SnO_2_; IMPS: intensity-modulated photocurrent spectroscopy; IMVS: intensity-modulated photovoltage spectroscopy; *J*_sc_: short-circuit current; *J-V*: photocurrent-voltage; PCE: photoelectric conversion efficiency; PVP: polyvinylpyrrolidone; SEM: scanning electron microscope; *V*_oc_: open-circuit voltage; XRD: X-ray diffraction; 1-D: one-dimensional; *τ*_d_: transit time; *τ*_n_: electron lifetime.

## Competing interests

The authors declare that they have no competing interests.

## Authors’ contributions

JND and NYN conceived and designed the experiments, and wrote the paper. YL carried out the experiments and took part in writing. HH and LB participated in the experiments. SZ participated in the discussion and correction of the paper. All authors read and approved the final manuscript.
